# Pediatric Migraine Aura Status and Cortical Laminar Necrosis: A Clinical Case and a Narrative Review

**DOI:** 10.31083/RN47684

**Published:** 2026-03-12

**Authors:** Federica Cernigliaro, Cristina Gallo, Giuseppina Alessi, Luca Maria Messina, Carola Meo, Edvige Correnti, Giuseppe Craparo, Vincenzo Raieli

**Affiliations:** ^1^Department of Health Promotion, Mother and Child Care, Internal Medicine and Medical Specialities “G. D'Alessandro”, University of Palermo, 90127 Palermo (PA), Italy; ^2^Neuroradiology Unit, Civico-Di Cristina – Benfratelli Hospital Palermo, 90134 Palermo (PA), Italy; ^3^UOS NPIA – ASP Trapani, 91016 Erice (TP), Italy; ^4^Child Neuropsychiatry Unit, Civico- Di Cristina -Benfratelli Hospital , 90134 Palermo (PA), Italy

**Keywords:** migraine, migraine aura status, cortical laminar necrosis, child, aura, stroke

## Abstract

**Introduction:**

Migraine aura status is a complication of migraine. It is characterised by the presence of at least 3 episodes of migraine aura within a 3-day period. The pain and/or associated symptoms are often debilitating. Cortical laminar necrosis (CLN) is a gyriform brain lesion caused by insufficient oxygen and glucose supply, resulting in the loss of cortical neurons, often due to cardiac arrest, global hypoxia, and hypoglycaemia. Metabolic disorders, hypoglycaemia, renal and hepatic dysfunction, and immunosuppressive chemotherapy. In children, cortical laminar necrosis has been linked to the subacute or chronic phase of brain damage due to hypoxic-ischaemic encephalopathy. Metabolic disorders, hypoglycaemia, renal and hepatic dysfunction, and immunosuppressive chemotherapy are included as other possible aetiologies. CLN has also been reported in patients with encephalitis, but it is extremely rare in migraine with or without aura.

**Clinical Case:**

We describe a 14-year-old boy with no previous neurological problems who was admitted to our unit due to the onset of acute and persistent symptoms characterised by headache, confusion, dysarthria, aphasia and visual disturbances. An initial emergency brain neuroimaging scan revealed edema localised in the supramarginal gyrus of the left cerebral hemisphere, with possible vascular etiology. A control magnetic resonance imaging revealed laminar necrosis of the cortico-pial area located in the same region.

**Conclusions:**

This clinical case is interesting due to the uncommon correlation between cortical laminar necrosis and migraine with aura (MA), the pediatric presentation, the location of hypoperfusion and the atypical progression of the migraine aura. We have reported a narrative review of the two disorders.

## 1. Introduction

Migraine is a disabling disease in pediatric and adult population and it is distinguished in Migraine Without Aura (MwA) and With Aura (MA). Rare complications of migraine include status migrainous, persistent aura without migraine infarction, and migraine infarction [1].

Migraine aura status is a rare complication of migraine attack. It is diagnosed by the presence of at least 3 episodes of migrainous aura over a period of 3 days. The pain and/or associated symptoms are often disabling. It was also confirmed in the appendix of the subsequent classification, International Classification Headaches Disorders 3rd (ICHD-3) of 2018, which modified the diagnostic cut-off, setting it at at least 3 aura episodes in 3 days. However, the proposed criteria are based on insufficient empirical evidence, especially for the precise cut-off in terms of the frequency of attacks required for diagnosis [2, 3].

Cortical laminar necrosis (CLN), also described as pseudolaminar necrosis (PLN), is a degeneration of neurons in the cerebral cortex in conditions where the supply of oxygen and glucose is inadequate to meet local needs. It can be observed during events that compromise the patient’s ability to supply the brain with sufficient nutrients to meet its needs [4].

A clarification must certainly be made on the terminology commonly used since the two definitions, CLN and PLN, although often used interchangeably, have different histological meanings: Cortical laminar necrosis involves all layers of the affected cerebral cortex, whereas pseudo laminar necrosis selectively affects the middle and deep layers [5, 6].

Here we describe a rare pediatric migraine aura status associated to the cortical laminar necrosis and report a narrative review of the two disorders.

Table 1 shows the diagnostic criteria of ICHD-3 [2] regarding the definition of Migraine Aura Status.

**Table 1.** **The diagnostic criteria ICHD-3 regarding the definition of Migraine Aura Status**.

Migraine Aura Status
ICHD-3 (2018)
Diagnostic criteria:
A. Migraine fulfilling criteria for 1.2 Migraine with aura or one of its subtypes
B. At least three aura episodes appearing within three days.
Comment: Other neurological disorders including reversible cerebral vasoconstriction syndrome, posterior reversible encephalopathy syndrome, and arterial dissection should be excluded by appropriate investigations.

ICHD-3, International Classification Headaches Disorders 3rd.

## 2. The Clinical Case

We present the clinical case of a 14-year-old male patient with a negative personal history of headache or neurological disorders, who came to our observation in the Pediatric Neuropsychiatry Unit, referred by the Emergency Department of the same hospital, for the onset of acute and persistent symptoms. While playing volleyball with friends, without any evident cranial or extracranial trauma, the boy experienced a sudden psychomotor slowdown, with aphasia and dysarthria. The mother, who rushed to provide first aid, reported marked pallor, sweating, and a blank and distant stare. He repeatedly touched the left side of his head and was unable to articulate words. During transport from his hometown to the emergency department and after triage, the state persisted, but aphasia gradually resolved. During hospitalization, in a pain-free phase of wellness, the patient described his experience to clinicians: “I felt like in a dream, I wanted to say something, but I couldn’t pronounce the words. I could hear what they were saying, but I didn’t understand. I was sweating and felt hot, like being inside a bubble because I couldn’t interact with the outside world, and I couldn’t communicate.” At that stage, he reported the onset of severe headache localized to the left parieto-occipital region and major visual function deficits, initially a right homonymous hemianopsia lasting about one hour, followed by transient blurred vision and phosphenes. He also reported disorientation and objective vertigo. On admission, general examination revealed a prostrated appearance, anxiety, and a tendency to touch the left side of the head due to a stabbing and pulsating pain. Neurological examination showed: normal cranial nerves, ocular motility, and coordination; positive Romberg with leftward deviation; mild right-sided weakness; no language disturbance at the time of formal assessment.

During inpatient stay, the patient experienced two further critical episodes. The first one (nighttime) was characterized by abrupt awakening with confusion, fear, disorientation, and failure to recognize roommate. He claimed that there were people in the room who were not really there (“there are doctors here, they have to operate on my ears”), suggesting a possible hallucinatory episode lasting a few minutes. Resolution was followed by severe left parieto-occipital headache and right upper limb paresthesias starting in the hand and extending proximally. The second episode (wakeful rest) was characterized by confusional state with possible phosphenes (“I see like when I rub my eyes”), lasting about 4 minutes, associated with paresthesias in the right hand. It was followed by a severe left parieto-occipital headache too, responsive to oral corticosteroid.

About six months before admission episodes were reported—at least two within two consecutive months—with poorly defined semiological characteristics, not clearly migrainous. During these episodes, the patient was described as agitated and frightened, with facial pallor, profuse sweating, and significant tachycardia with palpitations. During the second episode, nausea with vomiting was reported.

Due to the severity and sudden onset of symptoms, an urgent Brain computed tomography (CT) (General Electric Aascent FCE 128, Milano, Italy), was performed two hours after onset. It showed a millimetric hypodensity in the left parieto-occipital cortical-subcortical area, requiring further characterization.

During the second day of hospitalization, a contrast-enhanced brain magnetic resonance imaging (MRI) (Milano -Italy General Electric SIGNA EXPLORER 1.5 Tesla, GE Healthcare, Milano, Italy) was performed with T1weight (T1w) spin echo (SE), T2weight (T2w), Fast Spin Echo (FSE), Fluid Attenuated Inversion Recovery (FLAIR), Gradient Recalled Echo (GRE) and Diffusion Weighted Imaging (DWI) sequences.

**Fig. 1.**
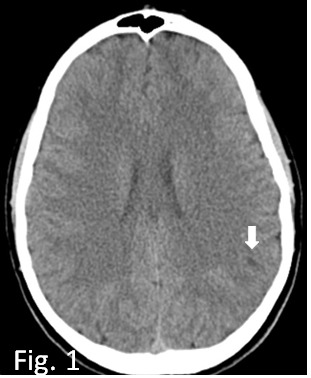
**Axial CT basal scan at the onset; slight millimetric hypodensity in parieto-occipital region (white arrow)**. CT, computed tomography.

**Fig. 2.**
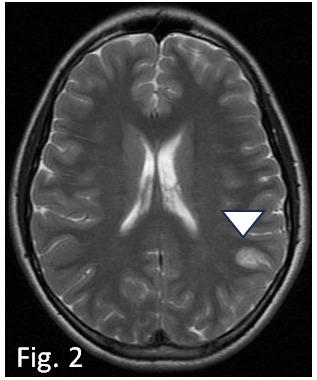
**Axial FSE T2W MRI performed one day after the debut, shows swollen edematous appearance of the cortex of the left supramarginal gyrus (arrowhead)**. FSE, Fast Spin Echo; MRI, magnetic resonance imaging.

**Fig. 3.**
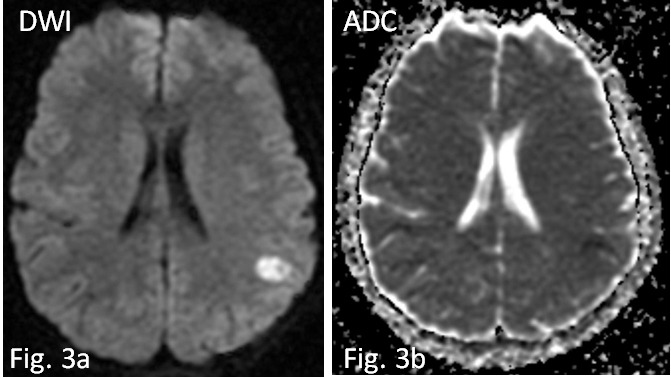
**In diffusion weighted imaging/apparent diffusion coefficient (DWI/ADC) sequence the lesion shows restriction of water molecule diffusivity due to cytotoxic edema (a,b)**.

**Fig. 4.**
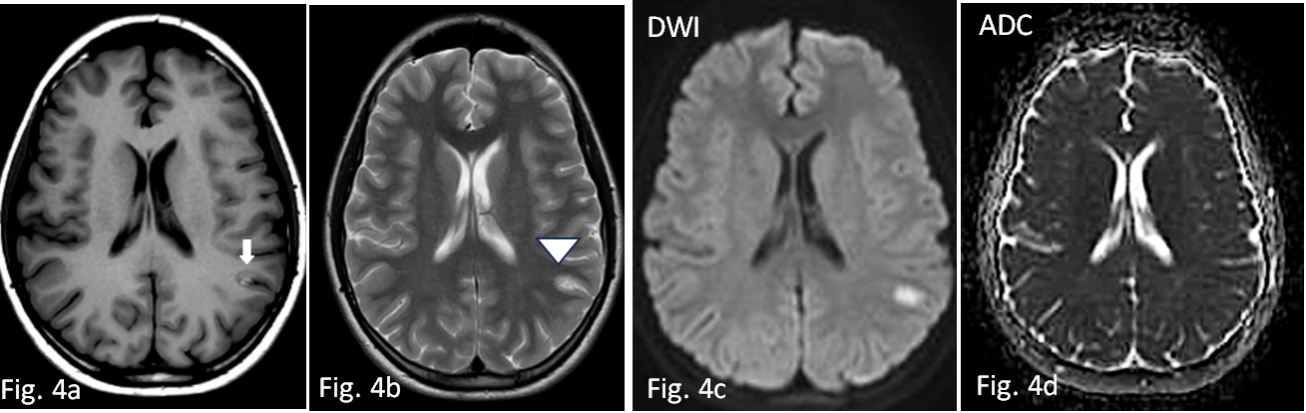
**Control MRI examination performed twenty days later**. Axial SE T1W basal image (4a) and axial FSE T2W (4b) respectively show appearance of cortical hyperintensity, suggestive of laminar necrosis (white arrow) and edema reduction (arrowhead). Hyperintensity is slightly reduced but still appreciable in DWI (4c), in the absence of signs of restriction in ADC (4d). SE, spin echo.

It showed a slight cortical thickening and signal alteration of the left supramarginal gyrus characterized by hyperintensity in T2/Flair sequences, diffusion weighted imaging (DWI)/apparent diffusion coefficient (ADC) restriction and no post-contrast enhancement (see Figs. 1,2,3,4). Moreover, a 3D time-of-flight (TOF) angio sequence was also performed to evaluate the intracranial arterial circulation showing no abnormalities.

These results were compatible with cytotoxic edema of uncertain pathogenesis (vascular, epileptic, or inflammatory).

Based on the clinical presentation, diagnostic hypotheses included: migraine with aura, epileptic seizure, acute cerebrovascular event, or infectious meningo-encephalitic condition. Toxicological urine screening was negative, excluding substance abuse. Clinical and electroencephalogram (EEG) findings, along with the absence of semiological features typical of seizures, excluded an epileptic pathogenesis. Similarly, the absence of systemic inflammatory signs, meningeal irritation, EEG abnormalities, or imaging evidence made meningoencephalitic or infectious causes unlikely. Serological tests for neurotropic viruses and neuroimmunological markers were also negative, excluding viral or immune-mediated encephalopathies. Due to the onset of hemiparesis during aura attacks, sporadic FHM could also be suspected, but genetic testing was not performed, both because there were no other relatives with a suspicion of hemiplegia and because one of the separated parents lived far away.

Follow-up brain MRI, performed twenty days later and compared with prior exam, showed reduced T2W signal alterations in the left parietal cortex and no restriction in DWI/ADC sequences. At the site of the lesion, mild hyperintensity was appreciated on T1W on the external cortical surface of the supramarginal gyrus, compatible with laminar necrosis.

Table 2 shows a clinical case summary (**Supplementary Material-Care checklist**).

**Table 2.** **Clinical case report summary**.

Patient	Male adolescent, first-year high school student
Background	Full-term birth after an uneventful pregnancy and elective C-section. Normal psychomotor and social development. Regular vaccinations. Hypersensitivity to erythromycin. No significant past medical or surgical history except for recurrent upper respiratory and gastrointestinal infections in early childhood.
	Positive family history of headache and migraine with aura and arterial hypertension.
Presenting complaint	Sudden-onset neurological episode during physical activity (volleyball): psychomotor slowing, aphasia, dysarthria, pallor, diaphoresis, and blank stare, followed by severe left parieto-occipital headache and transient right homonymous hemianopsia.
Clinical course	- Emergency phase: gradual resolution of aphasia, onset of intense headache, and visual deficits.
	- Hospitalization: two further paroxysmal episodes:
	• (1) Nighttime awakening with confusion, disorientation, visual hallucinations, followed by severe left parieto-occipital headache and right upper limb paresthesia;
	• (2) Daytime confusional state with phosphenes and right-hand paresthesias, again followed by severe headache.
	- Recurrent headaches during admission, sometimes localized, sometimes diffuse, responsive to NSAIDs or corticosteroids. The patient also reported transient memory difficulties and right-sided paresthesias.
Examination findings	- Prostrated appearance, anxious, left-sided headache.
	- Normal cranial nerves, ocular motility, and coordination.
	- Positive Romberg with leftward deviation.
	- Mild right-sided weakness.
	- No language disturbance at the time of formal assessment
Investigations	- CT brain: cortical-subcortical left parieto-occipital hypodensity.
	- MRI brain (with 3D TOF angio sequences): signal alterations in the left supramarginal gyrus consistent with cytotoxic edema; possible vascular, epileptic, or inflammatory etiology. Normal intracranial arterial circulation.
	- EEG: no epileptiform discharges; nonspecific slowing in left center-posterior regions.
	- Blood work: normal metabolic, infectious, autoimmune, and coagulation profiles.
	- Cardiac and nephrology investigations: normal echocardiogram, ECG, Holter monitoring, and renal evaluation.
	- Toxicology: negative.
Diagnosis	Acute cerebrovascular event associated with migraine with aura
Management & Outcome	Prophylactic anti-migraine therapy and physiotherapy for mild right-sided weakness.
	- At discharge: alert, oriented, mild residual hemibody weakness, no headache.
	- One-month follow-up: no new migraine episodes, persistent mild right-sided weakness. Control MRI showed improvement of parietal cortical lesions, with minimal laminar necrosis at the supramarginal gyrus.

NSAIDs, Non Steroidal Anti-Inflammatory drugs; TOF, time of flight; EEG, electroencephalogram; ECG, electrocardiogram.

The authors declare that written informed consent was obtained for publication.

## 3. Literature Review

Clinical semeiology of our case is strongly suggestive of migraine aura status, an infrequent condition that must be distinguished from hemicranius status [2] based on clinical differential diagnostic parameters, and the occurrence of vascular damage, radiologically demonstrated and described as laminar cortical necrosis.

It seems appropriate to describe the characteristics of these manifestations and to review the literature, both in general and in developmental age, regarding the possible correlations between migraine and cerebrovascular damage, with specific attention to descriptions of clinical cases in which the brain damage takes on the anatomopathological characteristics of laminar cortical necrosis. Furthermore, because of the presence of recurrence of migraine aura status within a short period of time in the patient in our study, a review of the literature regarding migraine aura status appears useful.

The literature review was conducted by searching Medline/PubMed and the Cochrane Library from 1980 to 2025, using the following terms: “Migraine Aura Status”, “Aura Status”, “Migrainous Infarction”, “laminar Necrosis”, “Cortical laminar Necrosis”, “Cortical laminar Necrosis and Migraine”.

### 3.1 Laminar Cortical Necrosis

CLN, also described as pseudolaminar necrosis (PLN), is a degeneration of neurons in the cerebral cortex in conditions where the supply of oxygen and glucose is inadequate to meet local needs.

The two definitions cortical laminar necrosis and pseudolaminar necrosis, have distinct histological meanings. Both terms are frequently misused for a wider range of ischaemic events that cause areas of intrinsic cortical T1 hyperintensity, cortical enhancement, or cortical dystrophic calcification [4].

Although the underlying condition of the cellular changes is presumably the same (i.e., liquefactive necrosis of the cortex, with monocyte influx and phagocytosis of cellular debris), damage limited exclusively to the cortex distinguishes laminar and pseudolaminar cortical necrosis from other more regional forms of ischaemic damage (e.g., thromboembolic cerebral infarction) [4].

More specifically it can be found in hypoperfusion of brain (widespread, secondary to cardiac arrest or hypotension, or localized, with a watershed distribution (border zone) due to hypotension or stenosis of the affected vessel (most frequently the internal carotid artery), hypoxia, hypoglycemia, hematological diseases such as severe anemia, and status epilepticus (as the brain requires increased glucose and oxygen supply).

CLN is a consequence of the greater metabolic activity of neurons compared to glial cells or adjacent white matter. Furthermore, not all of the cortex is equally vulnerable; rather, there is a selective vulnerability of certain layers of the cerebral neocortex (cortical layers 3, 4 and 5) to this metabolic stress, as well as certain cortical areas (e.g., the primary visual cortex and the perirolandic cortex) [4].

The selective vulnerability of grey matter also depends on the higher concentration of receptors for excitatory amino acids released after an anoxic-ischaemic event, as well as on higher metabolic demand.

Laminar necrosis can be observed a few hours after the onset of an anoxic-ischaemic event. Brain CT and MRI are mandatory tests for diagnosis. In the acute phase, DWI is superior to conventional MRI sequences in highlighting these cortical alterations [7].

The results of cortical laminar necrosis on CT may be subtle, such as gyroscopic alterations in attenuation, hypodense or hyperdense depending on the timing. During the acute phase of the ischaemic event, haemorrhages or calcifications are not usually detectable. After several days, an increase in the gyri will be evident, which can typically be observed for up to 3 months.

A brain MRI is absolutely necessary to proceed with the diagnosis. Although early cytotoxic edema induces a high signal on DWI with corresponding low ADC values in the affected cortex, and subsequently cortical enhancement, typically after 2 weeks, the intrinsic signal increase on T1 is the most specific MRI image [8].

The high T1 signal is believed to be due to the concentration of denatured proteins within necrotic neurons or lipid-filled macrophages; however, it is important to note that it does not represent the presence of calcium or haemorrhage. The curvilinear hyperintensity in T1 indicating laminar necrosis becomes evident as early as 3–5 days after the event, but usually reaches peak intensity after 2 weeks to 1 month, then slowly attenuates, usually over 3–8 months. Rarely, CLN remains detectable more than a year after the insult. The unaffected cortex may show increased signal or isointensity on T2-weighted images [8, 9].

### 3.2 Literature Review: Cortical Laminar Necrosis and Migraine

The existence of a complex relationship between headache and cerebrovascular diseases has long been known. Headache is a frequent symptom in children and adolescents, and, although rarely, it can be the presenting symptom of a stroke [10].

Preclinical and imaging studies highlight how an enhanced brain susceptibility to cortical spreading depression may be the candidate mechanism that increases the brain’s vulnerability to ischemia, thereby contributing to stroke risk in migraineurs [11, 12, 13]. The association between migraine with aura and ischemic stroke is well established [2]. In relation to the possible pathophysiology, it has been suggested that Cortical Spreading Depression can induce alterations in cerebral hemodynamic, increased vascular resistance, disruption of homeostasis, neuronal ion release, and the release of neuroinflammatory mediators. The dysfunctional endothelium may reduce the bioavailability of vasodilators and vasospasm, and induce a hypercoagulable state by increased oxidative stress. The depolarization waves of CSD induce a failure of brain ion homeostasis, efflux of excitatory amino acids, and increased energy metabolism, similar to the ischemic stroke or severe hypoglycaemia [14].

While the scientific literature is full of works that relate migraine to stroke or cerebrovascular damage in general, the situation changes drastically if we look for clinical descriptions of conditions in which the encephalic damage takes on the anatomopathological characteristics of laminar cortical necrosis. In fact, currently (September 2025), the search performed on PubMed brings to our attention only 6 works, none of which relate to the pediatric area:

- Familial Hemiplegic Migraine, Neuropsychiatric Symptoms and Erdheim–Chester Disease [15]: This article [15] describes the clinical case of a 51-year-old male with a history of familial hemiplegic migraine, which first appeared at the age of 18, complaining of approximately 6 attacks per year. The attacks were characterized by an initial scintillating scotoma, followed by unilateral throbbing headache of severe intensity. The headache was associated with nausea, vomiting and photophobia, along with ipsilateral weakness and sensory deficits lasting 5–6 days, with subsequent complete resolution. Upon recovery, he showed a visual field deficit and expressive language impairment. Brain MRI with contrast revealed hypervascularization and slight enhancement of the left hemisphere. FLAIR and T2-weighted images showed multiple diffuse lesions in the left cerebral cortex and cingulate gyrus. A follow-up MRI one month after the acute event showed a reduction of brain edema, but persistence and slight worsening of the lesions involving the left hemisphere, which took on the clear neuropathological appearance of cortical laminar necrosis. This case represents the only known correlation between familial hemiplegic migraine and cortical laminar necrosis.

- Migrainous infarction with appearance of laminar necrosis on MRI: this report describes the case of a 57-year-old woman. She complained MA attacks [16]. The migrainous attack started with photopsia in her left visual field over several minutes, associated with tinnitus, and mild left arm and leg paresthesias. Cortical laminar necrosis related to migrainous cerebral infarction [6]: This report describes a 29-year-old woman suffering from migraine episodes with visual and non-visual aura, which began a long time ago. During a typical migraine attack with aura, the clinical picture presented dysarthria, left hemiparesis and hemipesthesia, more pronounced in the upper limb, associated with brief, rapid and self-limiting ipsilateral facial movements. Four hours after onset, only headache and focal sensory-motor deficit persisted, and by day seven, the patient had fully recovered. Brain MRI performed 20 days after onset showed a subacute ischaemic lesion in the right temporoparietal cortex consistent with cortical laminar necrosis (CLN).

- Cortical laminar necrosis in a case of migrainous cerebral infarction [17]: This report describes a 27-year-old woman with migraine who was undergoing chronic treatment with oral contraceptives. The patient visited her doctor complaining of a severe migraine episode with persistent visual aura, which lasted until late at night. The following morning, the headache persisted and the patient developed a sudden episode of dysarthria, with right hemiparesis. About two weeks later, during another severe migraine-like headache, the patient’s symptoms worsened, and she reported right hemiparesis. She was admitted to hospital for stroke and underwent further radiological examinations: a brain MRI revealed a left temporal-parietal lesion, diagnosed as CLN.

- The case of a 37-year-old Caucasian female with migraine with visual aura that began at age 18. The visual aura presented with a scintillating scotoma followed by headache along with photophobia, nausea, and left homonymous hemianopia. Each episode lasted less than 30 minutes and subsequently resolved completely. The left superior homonymous quadrantanopia persisted. A brain MRI performed one month after a migraine attack revealed increased cortical signal with a gyriform appearance on T1-, T2-, and FLAIR images in the occipitotemporal regions. After contrast injection, cortical enhancement was observed in the same regions, and vascular study showed reduced perfusion. These findings were defined as compatible with cortical laminar necrosis [18].

- Cortical laminar necrosis as an initial manifestation of migraine in an apparently normal patient: This case report [19] describes the case of a 27-year-old woman with migraine with aura, who displayed a similar clinical phenotype to case 4 [17].

However, brain MRI revealed a different localization of the damage. Left frontoparietal hyperintensity with attenuated inversion recovery on T2/T1/fluid without diffusion restriction on diffusion-weighted imaging and subtle blooming on gradient echo were highlighted. These neuroradiological findings were consistent with cortical laminar necrosis.

In Table 3 (Ref. [6, 15, 16, 17, 18, 19]) we report the cases report of Cortical Migraine Necrosis and migraine cited in literature.

**Table 3.** **Literature review of Cortical laminar necrosis and Migraine; case reports**.

Author	Year	Gender	Status onset	Type of migraine
Black D. F. *et al*.	2004	M	51 years old	Familial hemiplegic migraine
Headache [15]				
Liang Y and Scott TF	2007	F	57 years old	Migraine with aura
Clin Neu Neurosurg [16]				
Arboix A. *et al*.	2013	F	29 years old	Migraine with aura
World J Clin Cases [6]				
Khardenavis V. *et al*.	2018	F	27 years old	Migraine with aura
BMJ Case Rep [17]				
Morais R. *et al*.	2018	F	37 years old	Migraine with aura
Clin Neurol Neurosurg [18]				
Sharma SR *et al*.	2019	F	27 years old	Migraine with aura
J Neurosci Rural Pract [19]				

M, male; F, female.

### 3.3 Literature Review: Migraine Aura Status

Migraine auras may rarely exhibit unusual temporal patterns, manifesting as recurrent attacks within a short period of time. This condition, termed “Migraine Aura Staus” was first mentioned by Haas [20] more than 30 years ago and described as aura episodes that occur “…several times in succession for several days, like a ‘burst’, and then disappear only for days or weeks, only to recur in another burst…”

As pointed out by some authors [21, 22, 23, 24], this syndrome should not be confused with status migrainosus, a debilitating migraine in which the headache lasts more than 72 hours (with or without aura), with persistent aura without infarction, nor with syndromes characterized by probable migraine with prolonged aura, since in these conditions there are no aura-free intervals. Migraine auras often manifest with visual and sensory symptoms, not always followed by headache, and their presentation can be confused with epileptic conditions or transient ischemic attacks (TIAs), particularly in elderly patients with additional sensory symptoms or speech impairment.

A literature review was conducted by searching Medline/PubMed and the Cochrane Library using the terms “aura state”, “repeated or recurrent migraine aura”, and “repeated or recurrent aura attacks”. Using the above search terms, we found only nine articles (nine case reports) [20, 21, 25, 26] describing patients with a large number of consecutive migraine auras in a short period of time. Only nine patients met ICHD-3 criteria, without underlying medical conditions. These patients, in fact, suffered from migraine with aura and had reported a sudden increase in the frequency of predominantly visual auras. In none of them, both physical examination and neuroimaging revealed pathological findings.

The main characteristics of these patients are summarized in Table 4 (Ref. [20, 21, 25, 26]).

**Table 4.** **Literature review of migraine aura status**.

Author – Year	Case nr	Gender	Status onset	Type of aura	Aura frequency
Haas, 1982	1	M	70 years old	Visual, sensorial	Intermittent (multiple aura episodes per day for 5 weeks)
Ann Neurol [20]					
Haas, 1982	2	M	18 years old	Visual	Up to 100 per day, for 8 weeks
Ann Neurol [20]					
Haan *et al*., Neurology 2000 [21]	3	F	<30 years old	Visual	More than 2 for at least 5 days
Haan *et al*., 2002	4	F	50 years old	Visual	Not reported
Headache [21]					
Haan *et al*., 2002	5	M	53 years old	Visual, sensorial	Not reported
Headache [21]					
Haan *et al*., 2002	6	F	23 years old	Visual	2–4 days for 2 weeks
Headache [21]					
Cupini and Stipa, 2007 Cephalalgia [25]	7	M	57 years old	Visual, sensorial	Not reported
Cupini and Stipa, 2007	8	F	28 years old	Visual, sensorial	3 per day for 10 days
Cephalalgia [25]					
Reinecke and Silberstein, 2007	9	M	60 years old	Visual	Up to 8–10 a day for a few weeks
Headache [26]					

## 4. Discussion 

The diagnostic evaluation of this patient was guided by the need to exclude acute conditions capable of producing this complex neurological phenotype.

We suggest a migrainous origin. Supporting factors included a first-degree family history of migraine, frequent symptomatic recurrences without interictal manifestations, and the absence of permanent deficits or cognitive decline. The age of the minor, the absence of general and cardiac risk factors favouring an ischemic lesion, the associated severe headache, and the reported march of the migraine aura support, in our opinion, the hypothesis that the aura status induced the cortical laminar necrosis. However, the alternative hypothesis—that the onset of the ischemic lesion induced a cortical spreading depression which caused the appearance of the characteristic clinical symptoms—cannot be unequivocally excluded [27].

The case is noteworthy for its unusual aura presentation. Migrainous aura usually arises from cortical spreading depression (CSD) [28], typically beginning in the occipital cortex and spreading anteriorly, producing transient visual, sensory, and sometimes language symptoms [29]. However, it can also present with other rarer symptoms, which make it extremely fascinating to study. Viana *et al*. (2016–2017) [30, 31] highlight the potential clinical variability of this disorder.

In our patient the sequence and characteristics of symptoms varied across three distinct episodes:

- The first attack began with language impairment, suggesting a frontal onset (Broca’s area), followed by visual symptoms consistent with posterior spread;

- The second attack involved emotional changes and complex visual hallucinations, suggesting parietal–temporal involvement;

- The third attack more closely resembled a classic occipital-to-frontal progression.

This variability suggests that CSD is not restricted to an occipital onset, but may arise in extra-occipital regions, with multidirectional spread. Such phenomena have been described in isolated adult cases [32], for first Raieli *et al*. [33] have documented a small series of atypical aura in pediatric patients across multiple episodes.

Because the patient experienced three aura episodes within three days, he meets the ICHD-3 criteria for aura status. This entity is rarely reported and poorly characterized, with limited data on its association with cerebrovascular complications.

A second remarkable feature of this case was the cerebrovascular complication. The recurrent CSD likely induced a state of diffuse cortical hyperexcitability and hypermetabolism, creating a mismatch between metabolic demand and cerebral perfusion. This mismatch may more easily render vulnerable areas at the border between grey and white matter or at the transition between the occipital cortex and neighbouring areas [34]. The involvement of extraoccipital cortical areas in all six reported cases of CLN and migraine supports these pathophysiological considerations.

To our opinion, for first time a pediatric case has described in the literature and long-term follow-up remains essential to assess potential developmental impact.

Certainly, individual case reports do not allow for in-depth conclusions, especially when there are very few reports in the literature, and therefore, it is necessary to accumulate further data. However, the increased use of accurate functional MRIs, serialized over short periods of time, EEG spectral analysis, and other neurophysiological methods may provide further information in the future study of other similar cases [35].

## 5. Conclusions

This report therefore, highlights an unusual presentation of migraine with aura, complicated by cortical laminar necrosis in a pediatric patient. It underscores the need for greater awareness of atypical aura patterns, recognition of migraine aura status, and further investigation into the potential cerebrovascular risks associated with migraine in childhood.

So, the case appears interesting for:

- The absolutely infrequent finding of laminar cortical necrosis in a situation suggestive of a migraine aura state, with atypical aura;

- The unusual march of the aura;

- The site of hypoperfusion;

- The presentation of these clinical-anatomical features in the pediatric age.

- Finally, if a subject with confirmed migraine with aura experiences repeated episodes of migrainous aura in a short period of time, the clinician may consider performing or repeating neuroimaging tests for a possible onset of a clinical complication of migraine (Migraine infarction) or of secondary diseases [35].

As can be seen from the literature review, previously mentioned, to date, this patient represents the first ever described case of laminar cortical necrosis following migraine with aura in pediatric age.

## Availability of Data and Materials

No new data were created, and clinical data are unavailable due to privacy or ethical restrictions. The datasets used and analyzed during the current study are available from the corresponding author on reasonable request.

## Supplementary Material

Supplementary material associated with this article can be found, in the online version, at https://doi.org/10.31083/RN47684.
